# Sexuality research in India: An update

**DOI:** 10.4103/0019-5545.69243

**Published:** 2010-01

**Authors:** Om Prakash, T. S. Sathyanarayana Rao

**Affiliations:** Department of Psychiatry, Institute of Human Behaviour and Allied Sciences (IHBAS), Delhi - 110 095, India; 1 JSS University, JSS Medical College Hospital, M.G. Road, Mysore - 570 004, India

**Keywords:** Dhat syndrome, female sexual dysfunction, India, male sexual dysfunction

## Abstract

This review provides the available evidence on sexual dysfunctions in India. Most of the studies have concentrated on male sexual dysfunction and hardly a few have voiced the sexual problems in females. Erectile dysfunction (ED), premature ejaculation (PME) and combinations of ED and PME appear to be main dysfunctions reported in males. Dhat syndrome remains an important diagnosis reported in studies from North India. There is a paucity of literature on management issues with an emergent need to conduct systematic studies in this neglected area so that the concerns of these patients can be properly dealt with.

## INTRODUCTION

Human sexuality is inherently related to some of the social and public health problems in India. These problems may involve contraceptive use, child abuse, sex education, legal issues of homosexuality and AIDS. These health problems have a significant impact on existing health infrastructure and budget. These problems also need to look within the context of poverty, stressful living situations, diverse cultural belief systems, quackery, ignorance and inadequate health services. However, there is little recognition of how these health problems are related to human sexuality and their dysfunctions. There is a need to understand how sexual attitudes, beliefs, and values act and influence these problems. Our cultural perspective can also shape the experience and understanding of these disorders.[1] There is a need to research sexual experiences and dysfunctions, which further influence adult behavior patterns in India.

In this review, our aim is to present sexual dysfunction from the Indian perspective. Available data, based clinical studies from India, are reviewed and important findings highlighted. Our presentation is limited to sexual dysfunction only and paraphilias will be not discussed.

### Sexual dysfunction in males

One of the first literatures in male sexual dysfunctions was reported by Bagadia *et al*. (1959).[2] They observed ignorance, superstitions, fears and guilt feelings about sex as major areas of concern, and developed a method of educational group therapy for minor sex disorders, which involved a psycho-education including anatomy, physiology and mind-body continuum related to sexual disorders.

Bagadia and his colleagues (1972)[3] studied 258 male out patient of teaching hospital setting with sexual problems as main concerns. They found anxiety over nocturnal emission (65%) and passing semen in urine (47%) main problems in the unmarried group; while impotence (48%), premature ejaculation (34%) and passing semen in urine (47%) were common in married group. Anxiety state (57%), schizophrenia (16%) and reactive depression (16%) were common psychiatric diagnosable conditions in that sample.

Nakra and his colleagues (1977)[4] studied sexual disorders in 150 males attending psychiatric unit of a teaching general hospital. They reported that 9.2% of all patients seen had potency disorders. The commonest psychosexual disorders were impotence (acute onset 11.3%; insidious onset 24%), premature ejaculation (PME) (acute onset 10%; insidious onset 15.3%), Dhat syndrome (with impotence/PME 10.7%; without 10%) and apprehension about potency (18%). The wives of these patients showed either helpful or indifferent attitudes towards the problem of sexual dysfunction. The authors also concluded that PME is a state of hyper-sexual arousal.

Using the same cohort, Nakra and his colleagues (1978)[5] found that nearly 75% of the patients had practiced masturbation before developing potency disorders and nearly 43% had guilt associated with masturbation. The authors also found nocturnal emission and adolescent homosexual contacts in 95% and 16% of the subjects respectively and of these 69% and 39% respectively had associated guilt feelings. 64% of the subjects considered loss of semen harmful to health.

Kar and Verma (1978)[6] studied the sexual lives of 72 married psychiatric patients and compared with 80 married relatives or friends from same socio-cultural background. With regard to marriage, 63% of subjects with schizophrenia and 24% of manic-depressives were married after the onset of the illness; 48.5% of the patients failed to perform sexually on *suhag raat* (first honeymoon night after marriage) compared with 18.7% of the controls faced same problem. Premature ejaculation was reported in 48% of subjects in ‘patient group’ and 40% in controls. Erectile impotence was reported in 27% and 13% in ‘patient group’ and ‘control group’ respectively. 63.4% subjects from ‘patient group’ described their sexual relationship unpleasant as compared to only 2.5% from ‘control group’ considered unpleasant.

Kumar and his colleagues (1983)[6] conducted a study on 40 married male neurotics and 22 healthy controls from teaching hospital setting. They found that the sexual behavior of the neurotics was similar to healthy controls before the onset of illness. There was a significant decrease in the frequency of coitus, sexual satisfaction of self, perceived sexual satisfaction of the spouse and sexual adequacy.

Bagadia and his colleagues (1983)[7] used behavioral techniques to treat 26 married males with PME and secondary impotence; 58% patients improved with those techniques. Gupta and her colleagues (1989)[8] described the application of Modified Masters and Johnson technique in the treatment of sexual inadequacy in 21 married males. 76.2% patients showed improvement after this technique.

Avasthi and his colleagues (1994)[9] conducted an outcome study of 66 male patients with psychosexual dysfunction in the context of socio-demographic and clinical variables. Short term outcome (of one year duration) and long term outcome (of seven years’ duration) of those patients were recorded. Erectile dysfunction (ED), PME, and combination of ED and PME were reported by 30, 12 and 45% of subjects respectively. Dhat syndrome, with ED/PME, was reported by 9% of the subjects. Nearly 38% of the patients dropped out of the treatment (‘drop-out group’). At one year follow-up, nearly 44% of the patients perceived improvement (‘improved at one year group’), while rest did not (‘no change at one year group’). At the end of seven years, nearly 70% of the original 66 patients could be recontacted. Significantly, a greater number of subjects from the ‘drop-out group’ had active sexual dysfunction than other two groups. The study proved that improvement in the short-term outcome indicated favorable long-term outcome.

Verma and his colleagues (1998)[10] analyzed data on 1000 consecutive patients with sexual disorders attending the psychosexual clinic at the tertiary care setting. They found premature ejaculation (77.6%) and nocturnal emission (71.3%) frequent problems followed by a feeling of guilt about masturbation (33.4%), small size of the penis (30%) and erectile dysfunction (23.6%). Excessive worry about nocturnal emission, abnormal sensations in the genitals, and venereophobia was reported in 19.5%, 13.6% and 13% of patients, respectively.

A file review of 178 male patients with sexual dysfunction by Avasthi and his colleagues (2003)[11] revealed that high income, married status, presence of partner at evaluation, and liberal attitude towards sexuality increased the chances of selection of behavioral sex therapy. The outcome of therapy was associated with treatment adherence. Participation of the spouse resulted in lower dropout rates.

Gupta and his colleagues (2004)[12] attempted to assess clinical profiles of 150 patients attending skin OPD for psychosexual problems. Among them, erectile dysfunction (34%) was the commonest problem, followed by premature ejaculation (16.6%), Dhat syndrome (15.3%), and nocturnal emission (14%).

Kendurkar and his colleagues (2008)[13] assessed the pattern of sexual dysfunction in the patients attending a marriage and sex clinic from 1979 to 2005 by looking into their medical records. After reviewing the data of 1242 patients, they found premature ejaculation being the most common complaint and the most commonly diagnosed clinical entity, followed by male erectile problems and Dhat syndrome.

### Sexual dysfunction in females

As compared to male sexual dysfunction, a few Indian studies are available in the area of female sexual dysfunction. This area remains largely unexplored. Agarwal (1977)[14] reported a study of 17 female cases of frigidity. All except one presented with neurotic or somatic symptoms. Frigidity was associated with ignorance regarding sexual activity, fear of pregnancy, marital disharmony, lack of emotional atmosphere, tiredness and poor precoital attention. Superficial psychotherapy and guidance helped 65% of the subjects with frigidity.

In the review by Kulhara and Avasthi (1995),[15] there was mention of one unpublished study from Chandigarh which documented 13 female patients out of 464 attenders of a special clinic dealing with marital and sexual dysfunctions. Vaginismus, dyspareunia and lack of sexual desire were the main problems reported.

Kar and Koola (2007)[16] conducted a postal survey among English-speaking persons from a south Indian town and found orgasmic difficulties in 28.6% females. Moreover, almost 40% of females reported to have never masturbated.

In the study among 100 consecutive women attending the Department of Pediatrics for the care of non-critical children in a tertiary care teaching hospital, Avasthi and his colleagues (2008),[17] found 17% of the subjects encountered one or more difficulties during sexual activities. These difficulties were in the form of headache after sexual activity (10%), difficulty reaching orgasm (9%), painful intercourse (7%), lack of vaginal lubrication (5%), vaginal tightness (5%), bleeding after intercourse (3%) and vaginal infection (2%). 14% subjects attributed these difficulties to their own health problems; further lack of privacy (8%), spouse’s health problems (4%) and conflict with spouse (4%) were the other cited reasons for those difficulties. None considered their sexual difficulty significant enough to demand a thorough clinical assessment.

In another cross-sectional survey of 149 married women in a medical outpatient clinic of a tertiary care hospital, Singh and his colleagues (2009),[18] reported female sexual dysfunction (FSD) in 73.2% subjects of the sample. The complaints elicited were difficulties with desire in 77.2%, arousal in 91.3%, lubrication in 96.6%, orgasm in 86.6%, satisfaction in 81.2%, and pain in 64.4% of the subjects. Age above 40 years and fewer years of education were identified as contributory factors. Women attributed FSD to physical illness in participant or partner, relationship problems, and cultural taboos but none had sought professional help.

### Dhat syndrome

Studies pertaining to Dhat syndrome, a culture bound syndrome, have mostly defined clinical features. This “semen loss”-related psychological distress has been extensively reviewed by Prakash (2007),[1] and Avasthi and Jhirwal (2005).[19] The important studies conducted in this area were done by Malhotra and Wig (1975),[20] Behere and Natraj (1984),[21] Singh (1985), Chadda and Ahuja (1990),[22] Bhatia and Malik (1991),[23] Perme *et al*. (2005),[24] and Dhikav *et al*. (2007).[25]

Wig (1960),[26] coined the term “Dhat syndrome,” characterized by vague somatic symptoms and guilt attributed to semen loss through nocturnal emissions, urine and masturbation though there is no evidence of loss of semen.

Behere and Natraj (1984)[21] and Bhatia and Malik (1991)[23] found that the patients with symptoms of Dhat syndrome were mostly young, recently married, poor, rural and from family with conservative attitudes towards sex. Most studies found that these patients lose their semen in sleep, with urine, masturbation, hetero/homosexual sex.

Most studies found erectile dysfunction (22-62%) and premature ejaculation (22-44%) as commonly associated psychosexual dysfunctions, while depressive neurosis (40-42%), anxiety neurosis (21-38%), somatoform/hypochondriasis (32-40%) as the most reported psychiatric disorders in patients diagnosed with Dhat syndrome.

Chadda and Ahuja (1990) could not find any abnormality on urine examination except oxaluria (10%) and phosphaturia (6%). On follow-up of these patients, Behere and Natraj (1984) found that majority of the patients recovered (66%), while the rest either improved (22%) or were unchanged (12%).

Behere and Natraj (1984)[21] and Bhatia and Malik (1991)[23] explored the patients’ beliefs regarding composition of Dhat; found majority believe semen, followed by pus, sugar, concentrated urine, infection or “not sure.” Majority considered masturbation and/or excessive indulgence in sexual activities as important causative factor, followed by venereal diseases, urinary tract infections, overeating, constipation or worm infestation, disturbed sleep or genetic factors.

Regarding management of Dhat syndrome, Wig (1960)[26] suggested emphatic listening, reassurance and correction of erroneous beliefs. Avasthi and Gupta (1997),[27] in their manual proposed that the management of Dhat syndrome involves sex education, relaxation therapy and medications.

Prakash and Meena (2007),[28] provided an explanation regarding this belief derived from the anatomy and physiology of penis. They proposed that patients with Dhat syndrome believe that whatever blood is collected in cavernous spaces during erection, probably converts into semen. Hence, with every sexual activity they lose blood; as blood is their source of energy, they lose energy everyday becoming more weak and lethargic.

Chadda and Ahuja (1990)[22] advocated psycho-education and culturally informed cognitive behavioral therapy. Bhatia and Malik (1991)[23] found anti-anxiety and antidepressant drugs better as compared to psychotherapy. Dhikav and his colleagues (2008)[25] advocated selective serotonin reuptake inhibitors along with regular counseling.

## CONCLUSION

This review highlights the available evidence in the field of psychosexual medicine in India. It is important to mention that all studies were from a hospital setting and none from community. Only a few studies explored female sexual dysfunction. Very few studies spoke about management issues. Dhat syndrome could be an important diagnostic entity to be researched. There is a strong need to perform studies in these areas.
